# Prevalence and risk factors of bovine tuberculosis in dairy cattle in Eritrea

**DOI:** 10.1186/s12917-016-0705-9

**Published:** 2016-05-25

**Authors:** Michael K. Ghebremariam, V. P. M. G Rutten, J. C. M. Vernooij, K. Uqbazghi, T. Tesfaalem, T. Butsuamlak, A. M. Idris, M. Nielen, A. L. Michel

**Affiliations:** Department of Infectious Diseases and Immunology, Faculty of Veterinary Medicine, Utrecht University, Utrecht, The Netherlands; Department of Veterinary Sciences, Hamelmalo Agricultural College, Keren, Eritrea; Department of Veterinary Tropical Diseases, Faculty of Veterinary Science, University of Pretoria, Pretoria, South Africa; Department of Farm Animal Health, Faculty of Veterinary Medicine, Utrecht University, Utrecht, The Netherlands; Veterinary Services, Ministry of Agriculture (MOA), Mendefera, Debub Region Eritrea; National Animal and Plant Health Laboratory, MOA, Asmara, Eritrea; Veterinary Services, MOA, Keren, Anseba Region Eritrea; Veterinary Services, MOA, Asmara, Maekel Region Eritrea

**Keywords:** Bovine tuberculosis (BTB), Prevalence, Comparative tuberculin test, Dairy, Eritrea

## Abstract

**Background:**

The prevalence of bovine tuberculosis (BTB) in dairy cattle in the three major milk producing regions of Eritrea was assessed by subjecting 15,354 dairy cattle, 50 % of Eritrea’s dairy cattle population, to the single intradermal comparative tuberculin test (SICTT). Skin test results were interpreted according to guidelines of the World Organization for Animal Health (OIE) with >4 mm as cutoff in skin thickness increase. In addition, we studied the relation between ‘physiological’ variables related to pregnancy and lactation, and the variable ‘region’ on the probability to be skin test positive.

**Results:**

The BTB prevalences at animal and herd levels were: 21.5 % and 40.9 % in Maekel, 7.3 % and 10 % in Debub, and 0.2 % and 1.6 % in the Anseba region, respectively. Overall, in the regions included, prevalence was 11.3 % (confidence interval (CI) 95 % CI, 11.29 – 11.31 %) and 17.3 % (95 % CI, 17.27–17.33 %), at animal and herd level, respectively. Considering positive herds only, the animal BTB prevalence was 36.8 %, 30.1 %, and 1.8 %, in Maekel, Debub and Anseba, respectively, and the overall animal prevalence within these herds was 32 %. In adult dairy cattle the probability of positive reactivity in the SICTT test was highest in pregnant animals as compared to the other categories.

**Conclusion:**

This study reports persistent prevalence of BTB as defined by positive SICTT in the dairy sector of Eritrea, especially in the regions of Maekel and Debub that are located in the central highlands of the country. To our understanding this is the first report that has encompassed all the major dairy farms in Eritrea and it will be instrumental in advocating future BTB control programs in the dairy sector.

**Electronic supplementary material:**

The online version of this article (doi:10.1186/s12917-016-0705-9) contains supplementary material, which is available to authorized users.

## Background

Bovine tuberculosis (BTB) is a chronic, infectious and contagious disease caused by *Mycobacterium bovis (M.bovis)* affecting cattle and other species. The disease in cattle appears both on intensive dairy farms and in extensive pastoral systems [1–5]. The standard diagnostic test used in our study and many others is the ‘single intradermal comparative tuberculin test’ (SICTT). This test has moderate to high sensitivity (68–95 %) and high specificity (96–99 %) [6–9]. *M. bovis* is zoonotic, and can be transmitted from animals to humans through consumption of raw milk, inhalation, and direct contact with saliva. Beside its zoonotic importance, BTB affects the livelihood of people in developing countries by compromising sustainable food supply, income and social status [10]. In Africa, for example, BTB is ubiquitous: about 85 % of the cattle and 82 % of human populations live in areas where BTB is either not or only partly controlled [11]. In 2004, BTB was reported by 26 of the 51 African countries who filed statistics with OIE [12].

BTB is present in Eritrea [13], where the integrated farming system includes a dairy cattle population of around 16,000 animals of exotic breed (Holstein Friesians (HF)) and crossbreds, 13,000 cattle of the indigenous Barka breed, and 1,500 animals of Sudanese breeds (MOA, Kahsay Negash, personal communication, 2010). BTB, first reported in Eritrea by Pirani in (1929), was reported to be highly prevalent in the capital city Asmara (Maekel region) and its surroundings in 2001 [13]. The BTB status in other regions of the country was never addressed.

Thus, the current study focused on the major milk producing regions (Maekel, Debub, Anseba), and its samples included more than 50 % of the total dairy cattle population of the country. It aimed to determine BTB prevalence and its association with various animal ‘physiological statuses’ related to pregnancy and lactation and ‘region’ in relation to positive reactivity in the SICTT throughout Eritrea’s dairy sector.

## Methods

### Study area

**Fig. 1:**
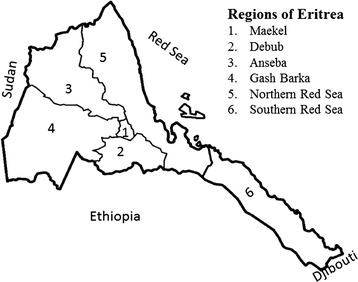
Map of Eritrea and the study areas. The map shows the six administrative regions of Eritrea and the three major milk producing regions of the country where the study was conducted: Maekel (1), Debub (2) and Anseba (3). Adapted from https://commons.wikimedia.org/wiki/File:Eritrea_regions_blank.png  (website re- visited: 06/05/2016)

Eritrea is located in the Horn of Africa and lies north of the equator between latitudes 12°22’ N and 18°02’ N, and longitudes 36°26’ E and 43°13’ E. It has an area of 124,400 square kilometres and is divided into six regions (zones) administratively, namely: Maekel, Debub, Anseba, Gash Barka, Southern and Northern Red Sea (Fig. 1), each of them comprising of several sub-regions. The country has diverse climatic zones. The mean annual temperature ranges from 16 °C in the highlands (around the capital Asmara) to extremely high temperatures (31 °C) in the lowlands. In the highlands, where the Maekel and Debub regions are located, the hottest months are usually May to June with the highest temperature reaching around 27 °C to 30 °C. Whereas in the western lowlands, where the Anseba region is located, the temperature ranges from 28 °C to 46 °C, except in December month when the temperature falls as low as 15 °C. The altitude ranges from 120 meters below sea level to 2400 meters above sea level in the central highlands. Dairy farms in the highlands of Eritrea are mainly concentrated in the Maekel and Debub regions [14], and in the lowlands the major dairy region is Anseba.

The livestock production system in the country is divided into three parts: intensive urban and peri-urban dairy husbandry system, extensive mixed crop-livestock system, and extensive pastoral system. The dairy farms are mainly located in and around the major towns of the regions. In Maekel the dairy farms are located in the capital Asmara and its environs. Dairy farmers in this region have shortage of land for housing, thus keep their dairy herds in confined areas in small houses. Most of the houses lack windows for adequate ventilation. In Debub, most of the dairy farms are located outside the major towns with few farms within the towns. The dairy farms outside the towns have adequate space for housing and grazing. Those dairy houses located in the towns have small sizes but with windows for ventilation. In this region dairy cattle are allowed to partially graze within the compounds of the farms. In Anseba, most of the dairy farms are located outside the major towns and have adequate spaces for partial grazing and housing. The dairy houses have adequate spaces and bigger windows. During the day the dairy cattle spend most of their time under shades outside the buildings.

In the mixed crop-livestock system, livestock are sedentary, kept in villages where grazing is communal. This production system is practiced mainly in the highlands (Maekel and Debub) and partially in Anseba (in the highland areas). In the pastoral system, large mobile herds are kept in the lowlands. Such husbandry system is mainly practiced in the western and eastern lowlands of the country, namely, in Gash Barka, Northern and Southern Red Sea regions, and partially in Anseba (Fig. 1). The herds in this production system come in contact with other herds during their movement. The largest proportion of the livestock (about 60 %) is kept in the lowlands under traditional system. The cattle population and its distribution in the six regions is shown in Table 1. The livestock species in these regions are predominantly small ruminants, cattle and camels.

**Table 1:** Official cattle population in 10^3^ in the six regions of Eritrea (Anonymous, 1997)

Region	Cattle population
Anseba	219
Debub	490
Gash Barka	917
Maekel	40
Northern Red Sea	178
Southern Red Sea	82
Total	1,927

### Study population and skin testing

In 2011 a cross-sectional survey was conducted to assess BTB prevalence in the three major milk producing regions, Maekel, Debub, and Anseba, in Eritrea (Fig. 1). All herds and all individual animals above six weeks of age were included in the study within the selected regions. The inclusion criteria were the same in all the regions. In total 15,354 dairy cattle in 3,149 herds were tested for BTB using the single intradermal comparative tuberculin test (SICTT). The survey was organized and coordinated by the Ministry of Agriculture, Eritrea.

The SICTT was used based on the guidelines of the OIE [6]. Two sites on the left side of the mid-neck, 12 to15cm apart, were shaved. The skin thickness was measured with a ‘Vernier caliper’ and recorded. The upper site was injected with 0.1 ml containing 2,500 IU/ml avian PPD (Instituto Zooprofilatico Sperimentale dell’ Umbria e delle Marche, Italia) using McLintock pre-set automatic syringes. Likewise, 0.1 ml of 2,500 UI/ml bovine PPD (Instituto Zooprofilattico Sperimentale deli Abruzzo e del Molise G. Caporale®, Italia) was injected into the lower site. Bovine PPD (PPD-B) and avian PPD (PPD-A) are defined as Purified Protein Derivatives of *M. bovis* and *M. avium*, respectively. All animals were ear tagged and their identities were confirmed at the time of reading. After 72 h, the skinfold thickness at the injection sites was re-measured by the same operator and with the same calliper used before. A skin reaction was considered positive when the skin thickness increase at the bovine site of injection was more than 4 mm greater than the reaction at the avian injection site. The reaction was considered inconclusive when the increase at the bovine site was between 1and 4 mm greater than the avian reaction. The reaction was considered negative when the increase in skin thickness at the bovine site was less than or equal to the increase in the skin reaction at the avian site of injection in the absence of clinical signs at the bovine injection sites. Moreover, information relating to age, sex, physiological status (respectively, bull (castrated/entire), heifer empty, heifer pregnant, lactating empty, lactating pregnant, dry pregnant) and region, was recorded for all the tested cattle (Table 2).

**Table 2:** Groups of cattle in different ’Physiological statuses’ and different age categories in the study (Eritrea, 2011)

Variables	Description
Calf	Young female/ male animal <2 years old
Bull	Male animal (entire/ castrated) ≥2 years old
Heifer empty	Young female animal ≥2 years old that hasn’t calved and is not pregnant
Heifer pregnant	Young female animal ≥2 years old that is pregnant and hasn’t calved before
Lactating empty	Mature ≥3 years old, lactating cow that is not pregnant
Lactating pregnant	Mature ≥3 years old, lactating and pregnant cow
Dry pregnant	Mature >3 years old pregnant cow that is approximately 4 to 8 weeks from calving, and not lactating
Age (three categories)	<3 years old
	3 to ≤5 years old
	>5 years old

### Data entry and analysis

The data was first entered in an Excel spreadsheet and checked for correctness by computing frequencies (pivot tables) using SPSS IBM version 20 software. Analyses of the association of the animals’ ‘physiological statuses’ on the prevalence of BTB as assessed by SICTT were performed using the statistical package R version 3.1.0 [15]. A ‘Generalized linear mixed model fit by maximum likelihood’ [16] was used with BTB as binary outcome (BTB present: yes or no) and with herd as random effect to account for correlated observations within herd. Explanatory variables were ‘region (forced in the model), ‘physiological statuses’ and ‘age category’ (the latter variable was included in the model on data of adult animals as ‘age’ is highly correlated with ‘physiological status’). In both models (on all animals and adult animals respectively) the final model contains ‘region and ‘physiological status.

The AIC (Akaike’s Information Criteria) selection criterion was used to select the ‘best model’. The analysis was performed in positive herds (at least one reactor animal) with ≥5 animals only (Model 1 & 2) (Tables 3 and 4). The variable ‘physiological status’ included calf, bull, heifer empty, heifer pregnant, lactating empty, dry pregnant, lactating pregnant. The variable ‘age’ included different age groups, namely; <3 years old, 3 to ≤5 years old, and >5 years old (Table 2).

**Table 3:** Model 1 ‘Physiological status’ and ‘region’ as potential risk factors for reactivity in the comparative tuberculin test on positive herds with ≥5 tested animals (4,776 observations within 344 positive herds) and animals tested, number and proportion of positive reactors in all positive farms (5269 observation). Estimated variance for herd: 1.118 on the logit scale

Physiological status and region	OR	95 % confidence interval		Number and % tested and % positive reactors in positive farms		
		Lower bound	Upper bound	Tested	% tested	% positive reactors
Calf (reference)	1.0			1613	30.6	14.6
Bull	5.2	3.3	8.3	147	2.8	30.6
Heifer empty	2.5	1.8	3.5	533	10.1	22.1
Heifer pregnant	5.8	4.2	8.1	460	8.7	34.4
Lactating empty	8.2	6.3	10.6	996	18.9	43.9
Lactating pregnant	10.8	8.4	13.9	1191	22.6	46.9
Dry pregnant	10.2	7.0	14.8	329	6.2	42.3
Anseba region (reference)	1.0			397	7.5	1.76
Debub region	8.5	1.9	37.1	1647	31.30	30.12
Maekel region	13.0	3.0	46.1	3225	61.21	36.84

**Table 4:** Model 2‘Physiological status’ as potential risk factor for reacting to tuberculin testing in adult dairy cattle in positive herds with ≥5 tested animals (2,356 observations in 326 herds) in Eritrea-2011, corrected for region. Estimated variance for herd: 1.245 on the logit scale

Physiological status	OR	95 % confidence interval	
		Lower	Upper
Bull (reference)	1.00		
Lactating empty	1.55	0.99	2.43
Lactating pregnant	2.24	1.43	3.50
Dry pregnant	2.01	1.19	3.40

## Results

### Descriptive analysis

Overall, 15,354 individual animals in 3,149 herds were tested in the three regions. The highest proportion of the cattle population under study was that of young stock <3 years (60 %) followed by mature animals 3 - ≤ 5 years (24 %). The prevalence of SICTT reactors was 11.3 % (95 % CI, 11.29 – 11.31 %) at individual animal level and 17.3 % 95 % CI, 0.1727-0.1733) at herd level. The individual animal prevalence would have increased by 0.4 % had we used single intradermal tuberculin test instead of SICTT. The number of individual animals and herds tested in each of the regions were: Maekel (*n* = 5,667 and 927), Debub (*n* = 6,827 and 1,839), Anseba (*n* = 2,860 and 383), respectively. The region with the highest animal and herd BTB prevalence was Maekel (21.5 % and 40.9 %), followed by Debub (7.3 % and 10 %) and Anseba (0.2 % and 1.6 %). Considering only the positive herds (*n* = 545) in the three regions, the total number of animals tested was 5,272. These comprised 3,226 animals in Maekel, 1,647 in Debub and 399 in Anseba. The BTB animal prevalence in the positive herds was 36.8 % (*n* = 1,188), 30.1 % (*n* = 496), and 1.8 % (*n* = 7) in Maekel, Debub, and Anseba, respectively. The reaction to bovine tuberculin test was strong with differences in skin thickness (PPD-B minus PPD-A) reaching 4.5 mm to 73 mm (Additional file 1). The additional file shows the bovine versus avian immune reactivity in the skin test in dairy cattle in Eritrea. This file will be useful as a baseline document for further studies in the area.

### Risk factors analysis

The highest prevalence of SICTT reactors was found in ‘lactating-pregnant cows (Table 3). In Table 3 the Odds ratios (OR), with 95 % confidence interval, of BTB status with animal group and region in positive farms with at least 5 tested animals are presented. The category ‘calf’ (animals of <2 years age) was taken as a reference for the ‘physiological status’ groups. Compared to calves animals in all different ‘physiological statuses’ groups were significantly more at risk of being skin test positive, with pregnant lactating cows being most at risk (Table 3, Model 1). Animals in Maekel were most at risk to be SICTT positive reactors, followed by those in Debub when compared to the Anseba region.

The odds ratios of BTB statuses of 2,356 adult animals in 326 positive herds with ‘physiological status’ as potential risk factor in relation to positive skin test results, taking ‘region’ into account are presented in table 4 (Model 2). Compared to bulls, as reference, females tend to have a higher risk, with pregnant animals significantly so.

## Discussion

The current study included more than half of the dairy cattle population of Eritrea and reports an overall prevalence of BTB at animal (11.3 %) as well as at herd (17.3 %) level. The individual animal BTB prevalence differed between the three regions included, ranging from 21.5 % in Maekel to 7.5 % in Debub and 0.2 % in Anseba. The BTB prevalence in skin test positive herds was not uniform; it was highest (37 %) in Maekel, followed by Debub (30 %), and Anseba (2 %).

The risk of having a BTB skin test positive animal was 13 times more likely in Maekel and ~9 times more likely in Debub than in Anseba (Table 3, Model 1). Differences in BTB prevalence may be due to risk factors varying across regions, as geographical location is in general an accepted risk factor. Herds and individual animal factors [17] may be attributed to the presence or absence of proper housing, adequate space for grazing and exercise, the type of breeds kept, and differences in climate.

Apart from differences in climatic circumstances between the regions the dairy housing system differs as well. In the highlands the farmers keep their animals indoors, in small houses with few or no windows [18], and as reported poor ventilation and housing may facilitate transmission of *M.bovis* [2, 19–21]. Close contact between animals is known to be a major risk factor as BTB is mainly transmitted via the respiratory route [22–24]. Similarly, close proximity of dairy farms and fence-line contact between animals, like in Maekel may be another important risk factor [25, 26]. These might have contributed over time to the persistence and high prevalence of BTB in the region, especially since no control program is in place in Eritrea. In contrast, in Anseba the dairy houses have adequate windows for ventilation and the animals spend more time outside in shaded areas and these factors might have contributed to the low number of SICTT reactors in the dairy farms of the region.

Asmara and its surroundings (Maekel region) was the first place in the country where exotic breeds were introduced for dairying. It was also in this area that BTB was reported for the first time in Eritrea, in 1929, and as already indicated in the previous study [13] as well as by the present study, the BTB prevalence is still on-going with an increasing trend. This might indicate that BTB has established in this region as it is likely for herd breakdowns to occur repeatedly in the same areas [27]. It may also be reasonable to argue that the subsequent development of an intensive dairy production system contributed to the establishment of *M.bovis* in this production environment as previously stated by various investigators [11, 28]. There is a good reason therefore to suspect that Maekel region might be acting as a source for *M.bovis* spread in the country, as it is a pioneering region for commercial dairying and a source of stocking and restocking of the dairy farms of the different regions in the country.

A major limitation of the present study was that the ‘breed’ of the animals tested was not sufficiently recorded by the operators during the skin testing period and thus could not be included in the analysis. However, we assume that the different breeds kept in the different regions of the country may have contributed to the relatively higher BTB prevalence observed in Maekel and Debub regions where the presence of the HF breed predominates at the farms as opposed to Anseba. Several studies have shown indigenous zebu cattle to be relatively tolerant to *M.bovis* infection as compared to exotic dairy breeds [3, 13, 20, 28–30]. To address this important aspect a relevant study is currently underway.

The present study indicated that pregnancy and lactation were significantly linked with the reactivity in SICTT. Lactating -pregnant cows were most at risk (OR ~ 11) to show positive reactions in the skin test compared to calves, and 2 times more compared to bulls. These results are in agreement with a similar study in Ethiopia [20] that reported that among ‘physiological status’ group, pregnant lactating animals had the highest prevalence of BTB. Kazwala et al., [31] also reported high reactivity in SICTT in lactating cows. Pregnant animals are reported to have two to threefold more severe inflammatory lesions with a rise to miliary tuberculosis, compared to non-pregnant young female adults [32]. It is difficult to explain the strongly increased odds ratio for SICTT positive responses in lactating-pregnant animals. Physiological alterations in immune responsiveness during pregnancy and lactation might influence skin test responsiveness and potentially lead to improved detectability of skin test positive females.

A case control study has shown that susceptibility of animals to BTB infection increases when they are fed deficient rations [33]. Currently, the available animal feeds in Eritrea are deficient quantitatively (inadequate in amount) as well as qualitatively (inadequate in their contents of carbohydrate, proteins, minerals, etc.) [34]. Feeding on such feeds might have contributed to the higher susceptibility to BTB in the pregnant cows as they are more demanding in their nutrient requirement.

Our risk factor analyses showed that ‘physiological status’ was strongly associated with SICTT positive reactivity (Table 4, Model 2). Since age and herd sizes have been indicated to be associated with the prevalence of BTB in several studies [13, 35–39], we included age and herd size as risk factors in the model. When the variable ‘age’ (3 levels) was added to the adult animal model on ‘physiological status’, age was not significant and did not influence the model estimates (results not shown). In the model with all age groups, calves clearly had the lowest risk of being SICTT positive as expected. Besides, including animals from all herd sizes, instead of only herds of 5 animals and above, did not have an important effect on the odds ratios of ‘physiological status’ vis-a-vis BTB prevalence (results not shown).

When compared to calves, bulls were at higher risk (OR ~ 5) to react to the skin test (Table 3, Model 1). The increased risk of bulls to be skin test positive as shown may be due to frequent contact of these animals with other herds during breeding, as sharing of bulls is a common practice in Eritrea [40], which is also considered to be a risk factor for BTB transmission between herds of animals [33]. In addition, in view of age, the potential cumulative exposure time for bulls is higher than for calves.

The overall results of the current study resemble those of similar studies in Ethiopia [1, 41–43]. The prevalences in Maekel were, however, higher than those reported by a study conducted previously by Omer et al. in the same region in Eritrea [10], which reported lower (14.5 %) animal, and comparable (41.7 % ) herd BTB prevalences. As expected the animal prevalence was higher than in the previous study since no BTB control measures were in place. The slightly higher herd prevalence in the previous study [10] might be due to the fact that they only considered animal herd sizes of >9 whereas in our study herd sizes of ≥1 were taken into account. Smaller herds have a lower prevalence [37, 39, 40].

In this study bacterial culture was not conducted due to the fact that skin test positive dairy cattle were not slaughtered after the disclosure of the results due to a lack of a compensation scheme.

In order to get more insight in the importance of BTB and its epidemiology in Eritrea, as a potential basis for a control program, further investigations in dairy cattle as well as in indigenous cattle, goats and camels kept under traditional livestock production systems are underway.

## Conclusion

The current study has brought to light the persisting prevalence of BTB as well as some animal characteristics, related to pregnancy and lactation, linked with SICTT positive skin test in the dairy sector in Eritrea. The BTB risk tended to vary by region and adult lactating-pregnant animals were the most likely to be test positive.

## Abbreviations

BTB, bovine tuberculosis; HF, Holstein Friesian; MOA, Ministry of Agriculture; NUFFIC, Netherlands University Foundation for International Cooperation; OIE, World Organization for Animal Health; *M.bovis mycobacterium bovis;* PPD, purified protein derivative; PPD-A, purified protein derivative for *mycobacterium avium;* PPD-B, purified protein derivative for *mycobacterium bovis;* SICTT, single intradermal comparative tuberculin test.

## Additional file

Additional file 1: Frequency of skin thickness (mm) after 72 h post inoculation in both the avian and bovine sites and the differences within the two (Bovine 72 h - Avian 72 h). The first two columns show the skin thicknesses on the avian site of injection after 72 h of the PPD-A inoculation and their frequencies (frq). The following four columns show the skin thicknesses on the bovine site of injection after 72 h of PPD-B inoculation and their frequencies. The following six columns show the skin thickness differences on the bovine sites versus avian sites of injection after 72 h of inoculations of both PPD-A and PPD-B (PPD-B minus PPD-A) and their subsequent frequencies. (XML 30 kb)

## References

[1] Regassa A, Tassew A, Amenu K, Megersa B, Abuna F, Mekbib B, Macrotty T, Ameni G (2010). Across-sectional study on bovine tuberculosis in Hawassa town and its surroundings, South Ethiopia. Trop Anim Health Prod.

[2] Radostits OM, Gay CC, Hinchcliff KW, Constable PD (2007). Diseases caused by mycobacterium species. Veterinary medicine- a text book of the diseases of cattle, sheep, pigs, goats and horses.

[3] Munyeme M, Muma JB, Samui KL, Skjerve E, Nambota AM, Phiri IGK, Rigouts L, Tryland M (2009). Prevalence of bovine tuberculosis and animal level risk factors for indigenous cattle under different grazing strategies in the livestock/wildlife interface areas of Zambia. Trop Anim Health Prod.

[4] Shirima GM, Kazwala RR, Kambarage DM (2003). Prevalence of bovine tuberculosis in cattle in different farming systems in the eastern zone of Tanzania. Prev Vet Med.

[5] Katale BZ, Mbugi EV, Karimuribo ED, Keyyu JD2, Kendall S, Kibiki GS5, Godfrey-332 Faussett P, Michel AL, Kazwala RR, van Helden P and Matee MI. Prevalence and risk factors for infection of bovine tuberculosis in indigenous cattle in the Serengeti ecosystem, Tanzania. BMC Vet Res. 2013;9:267. 10.1186/1746-6148-9-267 PMC3881215 24377705

[6] World Organisation for Animal Health (OIE): Manual of Diagnostic Tests and Vaccines for Terrestrial Animals. OIE, 6th Ed. OIE, Paris 2008; p.683-697.

[7] Monaghan ML, Doherty ML, Collins JD, Kazda JF, Quinn PJ (1994). The tuberculin test. Vet Microbiol.

[8] Goodchild AV, Downs SH, Upton P, Wood JLN, de la Rua-Domenech R (2015). Specificity of the comparative skin test for bovine tuberculosis in Great Britain. Vet Rec.

[9] Karolemeas K, de la Rua-Domenech R, Cooper R, Goodchild AV, Clifton-Hadley RS, Conlan AJK, Mitchell AP, Hewinson RG, Donnelly CA, Wood JLN, McKinley TJ (2012). Estimation of the relative sensitivity of the comparative tuberculin skin test in tuberculous cattle herds subjected to depopulation. PLoS One.

[10] Michel AL, Muller B, van Helden PD (2010). *Mycobacterium bovis* at the animal–human interface: A problem, or not?. Vet Microbiol.

[11] Cosivi O, Grange JM, Daborn CJ, Raviglione MC, Fujikura T, Cousins D, Robinson RA, Huchzermeyer HFAK, de Kantor I, Meslin FX (1998). Zoonotic Tuberculosis due to *Mycobacterium bovis* in developing countries. Emerg Infect Dis.

[12] Amanfu W (2006). The situation of tuberculosis and tuberculosis control in animals of economic interest. Tuberculosis.

[13] Omer MK, Skjerve E, Woldehiwet Z, Holstad G (2001). A cross sectional study of bovine tuberculosis in dairy farms in Asmara, Eritrea. Trop Anim Health Prod.

[14] Ghebremariam WK, Ortmann GF, Nsahlai IV (2006). A production function analysis of commercial dairy farms in the Highlands of Eritrea using ridge regression. Agrekon.

[15] R Core Team: R: a language and environment for statistical computing. R Foundation for Statistical Computing; 2014, Vienna, Austria. URL: http://www.R-project.org/.

[16] Bates D, Maechler M, Bolker B, Walker S. lme4: Linear mixed-effects models using Eigen and S4. R package version 1.1-7, 2014, URL: http://CRAN.R-project.org/package=lme4.

[17] Skuce RA, Allen AR, McDowell SW. Herd-level risk factors for bovine tuberculosis: a literature review. Vet Med Int. 2012;621210. 10.1155/2012/621210 PMC3395266 22966479

[18] Mogos A. Dairy Constraint Analysis in Eritrea, with special emphasis on Asmara and Surrounding Dairy Farms (Unpublished MSc thesis, University of Natal, South Africa), 2003

[19] Costello E, Doherty ML, Monaghan ML, Quigley FC, O’Reilly PF (1998). A study of cattle-to-cattle transmission of *Mycobacterium bovis* infection. Vet J.

[20] Elias K, Hussein D, Asseged B, Wondwossen T, Gebeyehu M (2008). Status of bovine tuberculosis in Addis Ababa dairy farms. Rev Sci Tech.

[21] O'Reilly LM, Daborn CJ (1995). The epidemiology of *Mycobacterium bovis* infections in animals and man: a review. Tubercl Lung Dis.

[22] Ameni G, Aseffa A, Engers H, Young D, Hewinson G, Vordermeier M (2006). Cattle husbandry in Ethiopia is a predominant factor affecting the pathology of bovine tuberculosis and gamma interferon responses to mycobacterial antigens. Clin Vaccine Immunol.

[23] Menzies FD, Neill SD (2000). Cattle-to-cattle transmission of bovine tuberculosis. The Vet J.

[24] Johnston WT, Vial F, Gettinby G, Bourne FJ, Clifton-Hadley RS, Cox DR, Crea P, Donnelly CA, Mc Inerney JP, Mitchell AP, Morrison WI, Woodroffe R (2011). Herd-level risk factors of bovine tuberculosis in England and Wales after the 2001foot-and-mouth disease epidemic. Int J Infect Dis.

[25] Kaneene JB, Bruning-Fann CS, Granger LM, Miller R, Porter-Spalding A. Environmental and farm management factors associated with tuberculosis on cattle farms in northeastern Michigan. JAVMA. 2002. 10.2460/javma.2002.221.837 12322923

[26] Munroe FA, Dohoo IR, Mc Nab WB, Spangler L (1999). Risk factors for the between-herd spread of *Mycobacterium bovis* in Canadian cattle and cervids between 1985 and 1994. Prev Vet Med.

[27] White PCL, Benhin JKA (2004). Factors influencing the incidence and scale of bovine tuberculosis in cattle in southwest England. Prev Vet Med.

[28] Vordermeier M, Ameni G, Berg S, Bishop R, Robertson BD, Aseffa A, Hewinson RG, Young DB (2012). The influence of cattle breed on susceptibility to bovine tuberculosis in Ethiopia. Comp Immunol Infect Dis.

[29] Ameni G, Erkihun A (2007). Bovine tuberculosis on small scale dairy farms in Adama Town, central Ethiopia, and farmer awareness of the disease. Rev Sci tech Off Int Epiz.

[30] Inangolet FO, Demelash B, Oloya J, Opuda- Asibo J, Skjerve E (2008). A cross-sectional study of bovine tuberculosis in the transhumant and agro-pastoral cattle herds in the border areas of Katakwiand Moroto districts. Uganda. Trop Anim Health Prod.

[31] Kazwala RR, Kambarage DM, Daborn CJ, Nyange J, Jiwa SFH, Sharp JM (2001). Risk factors associated with the occurrence of bovine tuberculosis in cattle in the Southern Highlands of Tanzania. Vet Res Commun.

[32] Weinberg ED (1984). Pregnancy-associated depression of cell mediated immunity. Rev Inf Dis.

[33] Griffin JM, Hahesy T, Lynch K, Salman MD, McCarthy J, Hurley T (1993). The association of cattle husbandry practices, environmental factors and farmer characteristics with the occurrence of chronic bovine tuberculosis in dairy herds in the Republic of Ireland. Prev Vet Med.

[34] Kayouli C, Tesfai T, Tewolde A. Country pasture/forage resource profiles, Eritrea FAO 2006

[35] Cleaveland S, Shaw DJ, Mfinanga SG, Shirima G, Kazwala RR, Eblate E, Sharp M (2007). *Mycobacterium bovis* in rural Tanzania: risk factors for infection in human and cattle populations. Tuberculosis.

[36] Cook AJC, Tuchili LM, Buve A, Foster SD, Godfrey-Faussett P, Pandey GS, McAdam KPWJ, 1996. Human and bovine tuberculosis in the Monze district of Zambia – a cross-sectional study. Br Vet J. 1996;152:37–46. 10.1016/s0007-1935(96)80084-4 8634864

[37] Griffin JM, Martin SW, Thorburn MA, Eves JA, Hammond RF (1996). A case–control study on the association of selected risk factors with the occurrence of bovine tuberculosis in the Republic of Ireland. Prev Vet Med.

[38] Humblet MF, Gilbert M, Govaerts M, Fauville-Dufaux M, Walravens K, Saegerman C (2010). New assessment of bovine tuberculosis risk factors in Belgium based on nationwide molecular epidemiology. JCM.

[39] Wright DM, Reid N, Montgomery WI, Allen AR, Skuce RA, Kao RR (2015). Herd-level bovine tuberculosis risk factors: assessing the role of low-level badger population disturbance. Sci Rep.

[40] Humblet MF, Boschiroli ML, Saegerman C (2009). Classification of worldwide bovine tuberculosis risk factors in cattle: a stratified approach. Vet Res.

[41] Ameni G, Ameni K, Tibbo M (2003). Bovine tuberculosis: Prevalence and risk factor assessment in cattle and cattle owners in Wuchale-Jida district central Ethiopia. J Appl Res Vet Med.

[42] Shitaye JE, Getahun B, Alemayehu T, Skoric M, Treml F, Fictum P, Vrbas V, Pavlik I (2006). A prevalence study of bovine tuberculosis by using abattoir meat inspection and tuberculin skin testing data, histopathological and *IS6110* PCR examination of tissues with tuberculous lesions in cattle in Ethiopia. Vet Med.

[43] Gumi B, Schelling E, Firdessa R, Aseffa A, Tschopp R, Yamuah L, Young D, Zinsstag J (2011). Prevalence of bovine tuberculosis in pastoral cattle herds in the Oromia region, southern Ethiopia. Trop Anim Health Prod.

